# Treatment for a rare case of phenazopyridine-induced methemoglobinemia: a case report and mini-review

**DOI:** 10.3389/fmed.2025.1568949

**Published:** 2025-05-08

**Authors:** Brendan M. Coyne, Syed M. Ishaq, Anjani Thota, Badal Thakkar

**Affiliations:** ^1^George Washington University School of Medicine and Health Sciences, Washington, DC, United States; ^2^Department of Internal Medicine, Sinai Hospital, Baltimore, MD, United States

**Keywords:** methemoglobinemia, phenazopyridine, methylene blue, ascorbic acid, COPD, hypoxia, case report

## Abstract

Methemoglobinemia is a condition caused by elevated levels of methemoglobin (MetHb) in the blood, a reduced form of hemoglobin that cannot properly bind to oxygen, interfering with delivery to tissues. If left untreated, this condition can be fatal. Phenazopyridine, an over-the-counter urinary tract analgesic, has been reported to cause methemoglobinemia in rare instances. In the present case, a 67-year-old patient demonstrated hypoxia and oral cyanosis in the setting of chronic phenazopyridine use and chronic obstructive pulmonary disease (COPD). A “chocolate-brown” coloration of his blood and an elevated MetHb level of 14.5% confirmed the diagnosis of methemoglobinemia. He was treated with methylene blue, ascorbic acid, bronchodilators, steroids, and supportive oxygen. This regimen led to a gradual improvement in the patient’s clinical condition, including his hypoxia, cyanosis, and MetHb levels. This report illustrates a rare, unique case of phenazopyridine-induced methemoglobinemia and acute hypoxic respiratory failure in a patient with pre-existing COPD. In such patients, effective management requires a careful treatment approach directed at both methemoglobinemia and COPD.

## 1 Introduction

Methemoglobinemia is a rare condition in which the iron species of hemoglobin (Hb) is oxidized into its ferric (Fe3+) state (1). This structurally transforms Hb into methemoglobin (MetHb), which is unable to bind to oxygen (O2), causing functional anemia due to the inability of red blood cells to deliver nutrients to target tissues throughout the body (1). The clinical presentation can range from minimal to severe, with some of the common symptoms including cyanosis, pallor, headache, and altered mental status (1). The condition is often fatal if MetHb levels exceed 70% in the blood (2).

Methemoglobinemia can be either congenital or acquired. Most cases are acquired and often occur in pediatric populations or adults with predisposing conditions (1). The most common offending agents identified in the general population include dapsone, primarily used as an antibiotic or anti-inflammatory, and benzocaine, which is used as a local anesthetic or as a “cutting” agent for street drugs (3). Other common inducers of methemoglobinemia include nitrates, nitrites, and sulfa drugs (4). Such medications can elicit methemoglobinemia by acting as oxidizing agents, converting Hb (containing ferrous iron) to its MetHb form (containing ferric iron) in the bloodstream (1).

Phenazopyridine is an azo dye that can be used as an adjuvant medication and analgesic for urinary tract infection (UTI) or irritation in the outpatient setting (5). It is a fairly common over-the-counter (OTC) medication with a good safety profile; however, it can rarely cause adverse effects, such as methemoglobinemia (6–17). Some cases of phenazopyridine-induced methemoglobinemia may additionally be accompanied by hemolytic anemia (18–22). These adverse effects are likely owed to phenazopyridine’s pharmacokinetics. Phenazopyridine is metabolized into aniline, which is an aromatic amine that can oxidize Hb’s iron groups (23). This can then cause methemoglobinemia and hemolytic anemia from oxidative stress (23).

The standard treatment for methemoglobinemia generally includes methylene blue, supportive treatment, and cessation of the offending agent (2). However, in patients with contraindications to methylene blue, such as those with glucose-6-phosphate dehydrogenase (G6PD) deficiency or concomitant serotonergic drugs (e.g., MAO inhibitors), alternate treatment options such as high-dose ascorbic acid may be considered instead (15, 16).

Phenazopyridine-induced methemoglobinemia is an overall rare occurrence, especially in adults without predisposing conditions such as G6PD deficiency. However, the condition is potentially life-threatening, and given widespread access to OTC phenazopyridine, providers and patients alike should be cognizant of the potential risk. Additionally, acquired methemoglobinemia from any cause should be considered a medical emergency in adults. Herein, we report the clinical presentation of acquired methemoglobinemia, discuss the underlying mechanisms, and review current guidelines and considerations for the management of this pathology.

## 2 Case presentation

A 67-year-old man initially presented to the emergency department (ED) with suprapubic abdominal pain. In the past year, he had been treated for recurrent urinary tract infections (UTIs) since placement of a suprapubic catheter, which was placed for his neurogenic bladder secondary to paraplegia caused by a gunshot wound. Each UTI was managed without complications, and the most recent UTI was successfully treated with ceftriaxone. To manage recurrent symptoms of urinary discomfort at home, the patient reported taking 200 milligrams (mg) of phenazopyridine three to five times per day for the past 6 months.

The patient also reported smoking two to three cigarettes per day and had a history of chronic obstructive pulmonary disease (COPD), for which he was prescribed inhaled budesonide-formoterol, tiotropium, and albuterol, but only adhered to albuterol. He also occasionally used 2 liters (L) of oxygen (O2) via nasal cannula (NC) at home. The patient’s medical history was also significant for essential hypertension, type 2 diabetes mellitus, and chronic pain. His home medications included amlodipine, atorvastatin, omeprazole, ergocalciferol, empagliflozin, nortriptyline, and oxycodone. He had no known history of recent travel, respiratory illness, or carbon monoxide exposure.

In the ED, the patient was incidentally found to have hypoxia. His present O2 saturation was 74% on room air (baseline saturation 97%), which only improved to 86% on 6 L via NC. He was afebrile, and other presenting vitals included a heart rate of 98 beats per minute (bpm) and a blood pressure of 121/83 millimeters of mercury (mmHg).

The patient’s physical exam revealed tachypnea and mild bilateral expiratory wheezing. There was bluish discoloration of the tongue, lips, and sclera. The patient was alert and oriented, and he exhibited no neurologic or psychiatric deficits aside from for baseline paraplegia. A chest X-ray in the ED showed left basilar opacity with a small left pleural effusion. Laboratory investigations demonstrated mild baseline anemia, as well as acute leukocytosis and combined respiratory acidosis and non-anion gap metabolic acidosis (Table 1). The metabolic acidosis was rapidly corrected with supportive treatment and intravenous (IV) hydration.

**Table 1:** Summary of the patient’s metabolic and blood work panels on the day of admission and discharge.

Laboratory value (reference range)	Units	Admission	Discharge
Sodium (135–145)	mmol/L	136	137
Potassium (3.5–5.0)	mmol/L	3.8	4.4
Chloride (95–105)	mmol/L	108	101
Bicarbonate (23–30)	mmol/L	20.1	29.4
Anion gap (7–16)	mmol/L	8	6
BUN (10–20)	mmol/L	13	18
Creatinine (0.7–1.3)	mmol/L	0.54	0.51
WBC (4–11)	K/μL	13.63	8.60
Hb (13.2–18)	g/dL	8.1	10.4
Total bilirubin (0.2–1.2)	mg/dL	1.0	0.7
G6PD Quant (127–427)	U	495	---

The patient was admitted to the medicine service for further management of acute hypoxic respiratory failure. A differential including but not limited to COPD exacerbation, pulmonary embolism, pneumonia, and drug toxicity was explored. The patient’s suprapubic pain and leukocytosis in the setting of his history of recurrent UTIs raised concern for a possible acute infection. However, a thorough workup, including urine analysis and multiple cultures, was negative for sepsis or other acute infectious processes. A computed tomography (CT) of the thorax showed no signs of consolidations or abnormalities, and a CT angiography of the chest was negative for pulmonary embolism. The X-ray and CT of the abdomen were also unremarkable.

Suspecting COPD exacerbation, the team initiated high-flow O2 therapy via NC at 40% fraction of inspired oxygen (FiO2) along with albuterol-ipratropium, budesonide-formoterol, and IV methylprednisolone. On this regimen, the patient remained hypoxic, despite denying dyspnea and feeling subjectively well respiratory-wise.

The persistent hypoxia in the mid-80s raised suspicion of methemoglobinemia. Blood was drawn for arterial blood gas (ABG), which revealed dark-brown blood, marked acidosis with a pH of 7.07, and a methemoglobin of 14.5% (Table 2).

**Table 2:** Summary of the patient’s blood gas analysis and co-oximetry results on the day of admission and discharge.

Laboratory value (reference range)	Units	Admission	Discharge
pH blood gas (7.35–7.45)	---	7.07	7.31
pCO2 blood gas (41–51)	mmHg	52	45
pO2 blood gas (75–100)	mmHg	283	83
HCO3 blood gas (22–29)	mmol/L	15	23
Lactate blood gas (0.5–2.0)	mmol/L	1.8	0.9
Carboxyhemoglobin (0–5 in smokers)	%	3.0	---
Oxyhemoglobin (90–95)	%	68.6	---
Methemoglobin (0–1.5)	%	14.5	5.8

With significant clinical clues from the patient’s medication history and presentation, a diagnosis of phenazopyridine-induced methemoglobinemia was made. The patient was administered one dose of methylene blue, with improvement in his cyanosis and a reduction in MetHb levels to 9.5%. The patient’s home nortriptyline was held that day. No additional doses of methylene blue were subsequently administered; the patient was instead treated with ascorbic acid so that nortriptyline could be continued. All other home medications were continued except for phenazopyridine, which was taken throughout admission.

On this treatment regimen, the patient’s clinical condition markedly improved. MetHb levels continued to drop to 5.8%, pCO2 decreased to 45 mmHg, and the pH stabilized at 7.31 (Table 2). With the patient’s O2 saturation consistently averaging at 98%, he was gradually weaned to room air. He was soon stabilized for discharge without complications and was transferred to an outpatient rehabilitation facility.

## 3 Discussion

Phenazopyridine is a commonly ingested OTC medication for the symptomatic relief of urinary pain, discomfort, and urgency. Although its use is typically recommended to be limited to two consecutive days, it is sometimes taken for prolonged durations, which can increase the risk of adverse effects. Our patient had been chronically ingesting phenazopyridine for 6 months, which was most likely responsible for his presentation of methemoglobinemia. The patient’s other home medications, including amlodipine, atorvastatin, omeprazole, ergocalciferol, empagliflozin, nortriptyline, and oxycodone, have not been shown to induce methemoglobinemia in the literature (4).

On NC, our patient denied subjective feelings of dyspnea, lethargy, or other symptoms, despite the presence of persistent hypoxia and tachypnea. This is not uncommon among other methemoglobinemia cases in the literature (17, 24). However, many such cases are also often resolved within hours of treatment, as opposed to our patient, who required multiple days of treatment (24). This prolonged duration may be owed to our patient’s prior history of COPD, for which he was not properly adhering to his home medications. The oxygenation status gradually improved upon a regimen of supportive O2, COPD medications, methylene blue, and ascorbic acid.

Our patient screened negative for G6PD deficiency, and he was thus not contraindicated to methylene blue administration. However, due to his concomitant nortriptyline, he was switched to ascorbic acid treatment to avoid serotonin syndrome.

It is important to note that in addition to methemoglobinemia, phenazopyridine also has the potential to induce sulfhemoglobinemia (25). Both conditions similarly cause hypoxia, and most available co-oximeters are unable to differentiate MetHb from sulfated hemoglobin (SulfHb). Thus, to ascertain a methemoglobinemia diagnosis, physicians must correlate co-oximetry readings with classic clinical signs, such as bluish discoloration and darkened blood and urine, as seen in our patient. Distinguishing methemoglobinemia from sulfhemoglobinemia is essential for guiding management. While methemoglobinemia is reversible with methylene blue or ascorbic acid, sulfhemoglobinemia would not respond to these treatments and may require a blood transfusion if severe enough (6, 25). The present case exemplifies the importance of astute history-taking and physical examination in guiding management of conditions such as methemoglobinemia.

## 4 Conclusion

Herein, we describe a case of acute hypoxic respiratory failure in a patient with phenazopyridine-induced methemoglobinemia and underlying COPD. In the workup of refractory hypoxia, providers should conduct a comprehensive review of all medications the patient is taking. This should include a discussion of ingested OTCs such as phenazopyridine. Phenazopyridine-induced methemoglobinemia should especially be considered in the setting of long-term use or increasing doses. When treating methemoglobinemia, medication reconciliation should be confirmed prior to methylene blue administration, given that this substance can interact with other medications (e.g., selective serotonin reuptake inhibitors, monoamine oxidase inhibitors), potentially causing serotonin syndrome. Additionally, a personal or family history of G6PD deficiency should be ruled out in patients receiving methylene blue. Ascorbic acid is a suitable alternative if methylene blue is contraindicated. Finally, when treating patients with methemoglobinemia, providers are encouraged to direct careful attention toward managing concomitant conditions, such as COPD.

## Data Availability

The original contributions presented in the study are included in the article/Supplementary material, further inquiries can be directed to the corresponding authors.

## References

[1] Ludlow JT Wilkerson RG Nappe TM. Methemoglobinemia. StatPearls. Treasure Island (FL): StatPearls Publishing (2024). Available online at: https://www.ncbi.nlm.nih.gov/books/NBK537317/30726002

[2] Iolascon A Bianchi P Andolfo I Russo R Barcellini W Fermo E. Recommendations for diagnosis and treatment of methemoglobinemia. Am J Hematol. (2021) 96:1666–78. doi: 10.1002/ajh.26340, PMID: 34467556 PMC9291883

[3] Ash-Bernal R Wise R Wright SM. Acquired methemoglobinemia: a retrospective series of 138 cases at 2 teaching hospitals. Medicine (Baltimore). (2004) 83:265–73. doi: 10.1097/01.md.0000141096.00377.3f, PMID: 15342970

[4] Curry S. Methemoglobinemia. Ann Emerg Med. (1982) 11:214–21. doi: 10.1016/s0196-0644(82)80502-7 7073040

[5] Eastham JH Patel P. Phenazopyridine. StatPearls. Treasure Island (FL): StatPearls Publishing (2024). Available online at: https://www.ncbi.nlm.nih.gov/books/NBK580545/

[6] Agrawal A Davis N Hsieh R Stallard S. Methemoglobinaemia with chronic phenazopyridine ingestion. BMJ Case Rep. (2019) 12:e227538. doi: 10.1136/bcr-2018-227538, PMID: 30772834 PMC6388894

[7] Koch AA Stolzenberg L Pathak PR Penot AR. Phenazopyridine-induced Methemoglobinemia: a case report. Cureus. (2023) 15:e33715. doi: 10.7759/cureus.33715, PMID: 36788851 PMC9922166

[8] Murphy T Fernandez M. Acquired methemoglobinemia from phenazopyridine use. Int J Emerg Med. (2018) 11:45. doi: 10.1186/s12245-018-0208-5, PMID: 31179913 PMC6326144

[9] Kc O Subedi A Sharma R Dahal PH Koirala M. A case of severe hypoxia caused by phenazopyridine-induced methemoglobinemia: a near fatal event from over-the-counter medication use. Clin Pract. (2022) 12:845–51. doi: 10.3390/clinpract12060089, PMID: 36412668 PMC9680236

[10] Shahani L Sattovia S. Acquired methaemoglobinaemia related to phenazopyridine ingestion. BMJ Case Rep. (2012) 2012:bcr2012006756. doi: 10.1136/bcr-2012-006756, PMID: 22987905 PMC4543562

[11] Gold NA Bithoney WG. Methemoglobinemia due to ingestion of at most three pills of pyridium in a 2-year-old: case report and review. J Emerg Med. (2003) 25:143–8. doi: 10.1016/s0736-4679(03)00162-8, PMID: 12901999

[12] Randazzo GP Ford EA Glauser FL. Methemoglobinemia caused by acute overdosage of phenazopyridine. West J Med. (1975) 122:427–9. PMID: 1130038 PMC1129765

[13] Mergey Devender H Patel P Abdelghany Y Grier W. A case of unexpected methemoglobinemia in the outpatient setting. Chest. (2023) 164:A6266–7. doi: 10.1016/j.chest.2023.07.4031

[14] Nasrullah A DiSilvio BE Malik KJ. Phenazopyridine-induced Methemoglobinemia: Dysuria or asphyxiation, choose wisely! C37. Case reports in drug induced lung disease. American Thoracic Society (2020) 201:A4945. doi: 10.1164/ajrccm-conference.2020.201.1_meetingabstracts.a4945

[15] Menakuru SR Dhillon VS Atta M Mann K Salih A. Phenazopyridine-induced methemoglobinemia in a Jehovah’s witness treated with high-dose ascorbic acid due to methylene blue contradictions: a case report and review of the literature. Hematol Rep. (2023) 15:325–30. doi: 10.3390/hematolrep15020034, PMID: 37367083 PMC10298695

[16] Hamza A Nasrullah A Singh R DiSilvio B. Phenazopyridine-induced methaemoglobinaemia the aftermath of dysuria treatment. Eur J Case Rep Intern Med. (2022) 9:003191. doi: 10.12890/2022_003191, PMID: 35265556 PMC8900564

[17] Stacy Amadife N Ul Alam D Whitesell PL. Uncommon, but not unseen: a potential cause of methemoglobinemia. Chest. (2023) 164:A6187–8. doi: 10.1016/j.chest.2023.07.3980

[18] Jeffery WH Zelicoff AP Hardy WR. Acquired methemoglobinemia and hemolytic anemia after usual doses of phenazopyridine. Drug Intell Clin Pharm. (1982) 16:157–9. doi: 10.1177/106002808201600212, PMID: 7075467

[19] Duran C Jalali E. Methemoglobinemia and anemia caused by pyridium overuse in an adult. Chest. (2017) 152:A387. doi: 10.1016/j.chest.2017.08.413

[20] Nathan DM. Acute methemoglobinemia and hemolytic anemia with phenazopyridine: possible relation to acute renal failure. Arch Intern Med. (1977) 137:1636–8. doi: 10.1001/archinte.1977.03630230104031, PMID: 921457

[21] Greenberg MS Wong H. Methemoglobinemia and Heinz body hemolytic anemia due to phenazopyridine hydrochloride. N Engl J Med. (1964) 271:431–5. doi: 10.1056/NEJM196408272710902, PMID: 14171812

[22] Zimmerman RC Green ED Ghurabi WH Colohan DP. Methemoglobinemia from overdose of phenazopyridine hydrochloride. Ann Emerg Med. (1980) 9:147–9. doi: 10.1016/s0196-0644(80)80270-8, PMID: 7362105

[23] National Research Council (US) Subcommittee on Acute Exposure Guideline Levels. Acute Exposure Guideline Levels for Selected Airborne Chemicals: Volume 1. Washington (DC): National Academies Press (US). (2000). 1, Aniline Acute Exposure Guideline Levels. 25057545

[24] Rathod BD Kamble N Awadhiya O Narang U Khot RS Kumbhalkar S. Shades of blue: a case series of acquired methemoglobinemia. Cureus. (2024) 16:e58312. doi: 10.7759/cureus.58312, PMID: 38752026 PMC11095280

[25] Askew SW Baranoski GVG. On the dysfunctional hemoglobins and cyanosis connection: practical implications for the clinical detection and differentiation of methemoglobinemia and sulfhemoglobinemia. Biomed Opt Express. (2018) 9:3284–305. doi: 10.1364/BOE.9.003284, PMID: 29984098 PMC6033548

