# Supplementary material for: Treatment for a rare case of phenazopyridine-induced methemoglobinemia: a case report and mini-review
Source: Front Med (Lausanne). 2025 May 8;12:1568949. doi: 10.3389/fmed.2025.1568949 (PMC12094986; doi:10.3389/fmed.2025.1568949)
Supplement: Supplementary file 1 [file Data_Sheet_1.pdf]

CARE
case report guidelines

CARE Checklist of information to include when writing a case report

(cc) BY-NC-ND

Topic	Item	Checklist item description	Reported on Line
Title	1	The diagnosis or intervention of primary focus followed by the words "case report"	55-62
Key Words	2	2 to 5 key words that identify diagnoses or interventions in this case report, including "case report"	87-89
Abstract (no references)	3a	Introduction: What is unique about this case and what does it add to the scientific literature?	69-72
	3b	Main symptoms and/or important clinical findings	74-76
	3c	The main diagnoses, therapeutic interventions, and outcomes	77-80
	3d	Conclusion—What is the main "take-away" lesson(s) from this case?	81-84
Introduction	4	One or two paragraphs summarizing why this case is unique (**may include references**)	120-133
Patient Information	5a	De-identified patient specific information.	133-134
	5b	Primary concerns and symptoms of the patient.	133-142
	5c	Medical, family, and psycho-social history including relevant genetic information	141-161
	5d	Relevant past interventions with outcomes	141-161
Clinical Findings	6	Describe significant physical examination (PE) and important clinical findings.	162-174
Timeline	7	Historical and current information from this episode of care organized as a timeline	n/a
Diagnostic Assessment	8a	Diagnostic testing (such as PE, laboratory testing, imaging, surveys).	162-166, 182-188
	8b	Diagnostic challenges (such as access to testing, financial, or cultural)	177-193
	8c	Diagnosis (including other diagnoses considered)	177-178, 194-200
	8d	Prognosis (such as staging in oncology) where applicable	242-243
Therapeutic Intervention	9a	Types of therapeutic intervention (such as pharmacologic, surgical, preventive, self-care)	200-204, 239-241
	9b	Administration of therapeutic intervention (such as dosage, strength, duration)	200-204, 239-241
	9c	Changes in therapeutic intervention (with rationale)	202-204, 239-241
Follow-up and Outcomes	10a	Clinician and patient-assessed outcomes (if available)	242-246
	10b	Important follow-up diagnostic and other test results	243-245
	10c	Intervention adherence and tolerability (How was this assessed?)	242-246
	10d	Adverse and unanticipated events	n/a
Discussion	11a	A scientific discussion of the strengths AND limitations associated with this case report	257-272, 302-305
	11b	Discussion of the relevant medical literature **with references**.	260-262, 265-268, 277-279
	11c	The scientific rationale for any conclusions (including assessment of possible causes)	268-282, 296-305
	11d	The primary "take-away" lessons of this case report (without references) in a one paragraph conclusion	309-327
Patient Perspective	12	The patient should share their perspective in one to two paragraphs on the treatment(s) they received	n/a
Informed Consent	13	Did the patient give informed consent? Please provide if requested	Yes ☒ No ☐
